# The impact of vector control on the prevalence of *Theileria cervi* in farmed Florida white-tailed deer, *Odocoileus virginianus*

**DOI:** 10.1186/s13071-019-3344-8

**Published:** 2019-03-13

**Authors:** Allison Cauvin, Karen Hood, Rebecca Shuman, Jeremy Orange, Jason K. Blackburn, Katherine A. Sayler, Samantha M. Wisely

**Affiliations:** 1 Department of Wildlife Ecology and Conservation, University of Florida, Gainesville, FL USA; 2 Florida Fish and Wildlife Conservation Commission, Gainesville, FL USA; 3 Spatial Epidemiology & Ecology Research Laboratory, Department of Geography, University of Florida, Gainesville, FL USA; 4 Emerging Pathogens Institute, University of Florida, Gainesville, FL USA

**Keywords:** Acaricide, Cervid, Hemoparasite, Piroplasm, *Theileria*, Tick-borne

## Abstract

**Background:**

Vector-borne diseases exert a global economic impact to the livestock industry. Understanding how agriculture practices and acaricide usage affect the ecology of these diseases is important for making informed management decisions. *Theileria cervi* is a hemoprotozoan parasite infecting white-tailed deer (*Odocoileus virginianus*) and is transmitted by the lone star tick, *Amblyomma americanum*. The purpose of this study was to determine if acaricide treatment decreased hematozoan prevalence in farmed white-tailed deer when compared to geographically-close wild deer or altered the genotypes of *T. cervi* present.

**Results:**

We compared prevalence of *T. cervi* in 52 farmed adult white-tailed deer which were regularly treated with permethrin and ivermectin, 53 farmed neonates that did not receive treatment for vector control, and 42 wild deer that received no form of chemical vector control. Wild deer had significantly higher prevalence of *T. cervi* than farmed deer. Additionally, no neonate fawns tested positive for *T. cervi*, and we found that age was a significant predictor of infection status. We found no difference in genotypic variation in *T. cervi* isolates between adjacent herds of farmed and wild white-tailed deer, although a divergent genotype X was identified. Chronic infection with *T. cervi* had no significant effects on mortality in the white-tailed deer.

**Conclusions:**

We found significantly lower prevalence of *T. cervi* infection in farmed (40%) compared to wild white-tailed deer (98%), which may be due to the inclusion of chemical vector control strategies. More work is needed to determine the implications, if any, of mixed genotypic infections of *T. cervi*, although we found no significant effect of infection with *Theileria* on mortality in farmed deer. *Theileria* infection does sometimes cause disease when an animal is stressed, immunosuppressed, or translocated from non-endemic to endemic regions.

## Background

Worldwide, vector-borne diseases cost billions of dollars in economic losses to the livestock industry annually [1]. The resurgence of many vector-borne protozoan infections, some of which are expanding into new geographical regions [2] or developing resistance to commercially used insecticides [3], has brought renewed attention to integrated control of vectors [4]. As a result, better characterization of the dynamics of vector-host-parasite systems and an understanding of the impact of vector control techniques on animals (both in livestock and wildlife) is crucial to ecosystem health. Variation in the success of vector control strategies across the global agro-ecological system [5] suggests that comparative or baseline investigations into the success of vector control in understudied farming systems can provide valuable information to the common global strategy of integrated pest management [6].

Tick-borne parasites in the genus *Theileria* are of concern in both managed and wild ruminants [7]. These obligate intracellular parasites are transmitted by ixodid ticks and have been detected in select mammalian species on almost every continent [8]. These organisms cause some of the most economically important and costly livestock diseases in the world [9]. However, the epidemiology of these parasites can vary widely. *Theileria parva*, the etiological agent of East Coast fever, can exist in a state of endemic stability in indigenous cattle breeds and cause little economic loss with little intervention or can cause enormous economic loss in naïve herds [5]. These epidemiological differences are driven by the prevalence of infection within herds and sympatric reservoir species, the age at which animals are infected, and management actions such as chemical vector control [10].

In North America, *T. cervi* has been observed in white-tailed deer (*Odocoileus virginianus*), elk (*Cervus canadensis*) and mule deer (*Odocoileus hemionus*) since at least the 1960s [11–13]. It is transmitted by the lone star tick *Amblyomma americanum* and represents a species that rarely causes clinical disease [14]. In cases where *T. cervi* has been linked to disease, the host was malnourished and co-infected with other parasites, or the host was translocated into an infected herd from a non-endemic area [15, 16]. Additionally, *T. cervi* was found to cause mortality in white-tailed deer fawns in Delaware, USA, and may be a more important parasite affecting the health of wild deer than previously thought, especially with the increase in abundance and range of the vector, lone star ticks [17]. Despite this, disease caused by *T. cervi* infection has not been well-documented.

The taxonomy of species within the genus *Theileria*, including *T. cervi*, is poorly defined [18, 19]. Considerable genetic diversity exists, and multiple genotypes are often considered variants of a single species because of similarities in their life history (same mammalian host and tick vector), overall pathogenicity, and genetics (i.e. they group within the same phylogenetic clade). Further complicating the taxonomy and epidemiology of these parasites are the *Theileria* species that are generally considered benign but occasionally cause disease due to a variety of factors including strain variation, as has been observed for *Theileria orientalis* [20], or due to increased host susceptibility caused by infection with other hemoparasites, as seen with *Theileria mutans* [21].

Few studies have explored *Theileria* prevalence and distribution in North American cervid populations. These studies are warranted due in part to the growing agro-industry for white-tailed deer production and the unexplored epidemiology of *Theileria* in this production system. A comparison of farmed and wild deer allows for an evaluation of vector control on farms, which have high densities of deer but are treated with acaricides and wild, free-ranging deer, which typically have lower densities but have limited vector management (i.e. prescribed fire) [22].

The objective of our study was to identify whether *T. cervi* prevalence varied in relation to host characteristics (age and sex) and management regimes (farmed and treated with acaricides or an untreated wild population). We sequenced multiple *T. cervi* genotypes in farmed and wild cervid populations to determine which genotypes were circulating in this region of Florida, USA. This study aims to provide deer farmers and land managers in North Florida with the baseline data necessary to develop effective integrated pest management plans to limit tick-borne diseases to managed herds and adjacent herds.

## Methods

### Sample collection

For a comparison of *T. cervi* prevalence and genetic variation in wild *vs* farmed deer, we sampled 52 farmed adult, 53 farmed neonate, and 42 wild adult white-tailed deer in Gadsden County, Florida between March 2016 and January 2017. To determine how age influenced the probability of infection and whether *T. cervi* could be vertically transmitted, farmed neonates were sampled within 24–48 hours of birth during the May-June 2016 fawning season. Farmed adult deer were sampled in March 2016 and again in September and November 2016 as part of routine handling. Farmed deer sampled in this study were maintained at densities of 5–15 animals per ≃0.8 ha pen in a 10-pen block. The block was adjacent to an approximately 200-ha hunting preserve stocked with white-tailed deer (not sampled in this study) and a variety of exotic cervid and bovine species, with roughly 1.5 animals/ha.

Farmed animals were treated with oral ivermectin (Durvet, Blue Springs, MO, USA) 3 times per year at a dosage of ~0.15 ml/kg of body weight, as self-reported by the farmer. Farmed animals also occasionally received injectable ivermectin at a dosage of 0.02 ml/kg, when available. Additionally, the pens were treated with a misted 75% Tengard SFR One Shot permethrin (UPI, King of Prussia, PA, USA) and 25% Exponent (MGK, Minneapolis, MN, USA) insecticide synergist solution. Exponent is a commercially-available insecticide synergist that contains the active ingredient piperonyl butoxide, which is often used in conjunction with pyrethroids to increase their effectiveness. This solution was applied in a twice-a-day, ‘two weeks on, one week off’ system during the summer months. The adjacent hunting preserve was also occasionally misted during summer.

During routine health checks performed by farm staff, adult deer were momentarily, mechanically restrained in chutes specifically designed for deer, and whole blood was collected by jugular venipuncture. All blood samples were collected in EDTA-coated vacutainer tubes (Beckton, Dickinson & Co., Franklin Lakes, NJ, USA), kept at 4 °C ≤ 72 h, and then stored at -80 °C prior to extraction of nucleic acids.

Wild white-tailed deer (*n* = 12) captured in May-June 2016 (spring 2016) were sampled as part of an ongoing movement study of deer on lands managed by Florida Forest Service and Florida Fish and Wildlife Conservation Commission, that were approximately 11 km from the farm in Gadsden County, Florida. The habitat in this area included natural upland pine stands, commercial pine stands and dense bottomland forests. These lands were typically utilized for a variety of working/recreation purposes and were managed heavily with prescribed fire. Prior to handling, wild animals were darted and chemically immobilized with BAM (butorphanol, azaperone, medetomidine combination, ZooPharm, Windsor, CO, USA). Additionally, hunter-harvested wild white-tailed deer (*n* = 42) were opportunistically sampled in January 2017 at a hunter check station located on the same managed lands as described above. These samples were included in the autumn 2016 wild cohort for further comparative analyses, as this was the closest time point to the farmed white-tailed deer sampling in September and November 2016. Blood was collected *post-mortem via* cardiac puncture from these animals, which were all male and > 1.5 years of age due to state harvest regulations. All blood specimens from wild deer were collected and handled identically as described above for farmed animals. Wild deer were not treated with acaricides.

### Molecular methods

We extracted DNA from whole blood samples using the Qiagen DNeasy Blood & Tissue Kit (Qiagen, Valencia, CA, USA) according to the manufacturer’s protocol. In order to genotype the *Theileria* species in our sampled cervids, we used a PCR assay designed to amplify the V4 hypervariable region of the *18S* rRNA gene. The primer set targets a region within a gene sequence amongst all previously sequenced *Theileria* species [8]: *Theileria* forward primer (TH FOR: 5′-TGA CAC AGG GAG GTA GTG A-3′) and *Theileria* reverse primer (TH REV: 5′-TCA GCC TTG CGA CCA TAC T-3′) [23]. We used 2 µl of template DNA in a 25-μl reaction that included 5 μl of Promega Green 5× Flexi Buffer (Promega, Madison, WI, USA), 1 μl each of the forward and reverse primers at 10 μM, 1.75 μl of 3 mM MgCl_2_, 2 μl of 2.5 mM dNTP (Invitrogen), and 0.25 μl GoTaq Flexi DNA Polymerase (Promega). The PCR thermal profile began with a 2 min initial denaturation step at 95 °C, followed by 40 cycles of 45 s at 94 °C, 45 s at 55 °C, and 1 min at 72 °C, with a final extension step at 72 °C for 5 min.

After gel electrophoresis, we visualized our PCR product by UV transillumination with RedView fluorescent gel stain (Genecopoeia, Rockville, MD, USA). We then randomly selected 4 positive samples and purified them using a QIAQuick PCR Purification kit (Qiagen) following the manufacturer’s protocol. The purified products were Sanger sequenced, and these sequences were identified as *T. cervi* by Basic Local Alignment Search Tool (BLAST) comparisons to the NCBI/GenBank database.

To identify common genotypes in farmed and wild deer populations, we randomly selected amplicons of the appropriate size amplified from wild deer (*n* = 5) and farmed deer (*n* = 4) matched by sex and age. We inserted these amplicons into MACH1-T1 competent cells using the TOPO TA Cloning Kit (Invitrogen, San Diego, California, USA). Using the PureLink Quick Plasmid MiniPrep Kit (Invitrogen), we purified five plasmid clones per sample and then obtained sequence information by Sanger sequencing. These were bidirectionally edited using FinchTV (FinchTV, Geospiza.com) and aligned using CLUSTALW [24].

### Phylogenetic analyses

Using the program Geneious version 9.0.5 (Biomatters Ltd., Auckland, NZ), we constructed a phylogenetic tree of *T. cervi* sequences detected in our study population using the Maximum Likelihood method (bootstrap = 1000 replicates) and determined evolutionary distances using the Tamura-Nei evolutionary model [25]. *Toxoplasma gondii*, a misnamed member of the microti group “*Theileria annae*” and *Theileria capreoli* were included to provide a better overview of how groups clustered within the *Theileria* lineage. We also included the 10 most similar sequences in GenBank determined by BLAST results for reference (AY735127.1, U97055.1, AF162433.1, U97056.1, AF086804.1, U97054.1, AY735135.1, AY735125.1, AY735133.1 and AY735132.1). Although there is a high level of microheterogeneity found within the V4 region of the *18S* rRNA gene, and sequences generated from this region are typically not ideal for phylogenetic studies, this gene remains the standard for diagnosis and analysis of genetic diversity of *Theileria* spp. [26, 27].

### Statistical analyses

We conducted all statistical analyses using R version 1.0.136 [28]. Univariate logistic regression was performed using the GLM function of the base *stats* package to evaluate age as a predictor of *T. cervi* infection status in farmed deer. Prior to testing the effect of treatment (wild or farmed deer) or sex on infection prevalence, we examined the data for a confounding effect of age. We compared infection status among wild and farmed yearling and adult deer using a t-test to investigate differences in average age between the farmed and wild white-tailed deer cohorts. We did not test for the influence of sex because adult farmed males (mean age of 1.15 years) were substantially younger than adult farmed females (mean age of 3.56 years). To explore whether there was seasonal variation in *T. cervi* prevalence, we compared prevalence between deer sampled in spring and autumn. To avoid any seasonal bias, only farmed and wild deer sampled within roughly the same season were used in this analysis. Differences between prevalence of *T. cervi* genotypes between farmed and wild deer were evaluated *via* Fisher’s exact test. All tests were considered significant at a *P*-value < 0.05. To determine if mortality rates were significantly different among infected and uninfected farmed individuals, we followed white-tailed deer survival outcomes ~18 months post-sampling, and calculated all-cause mortality rates based on the proportion of infected *vs* uninfected individuals that died. Additionally, samples from deceased animals were sent to the Cervidae Health Laboratory at the University of Florida for a cause of death to be determined. Odds ratios and *P*-values were calculated according to [29, 30], respectively, using MedCalc online software [31].

## Results

### Prevalence in wild *vs* farmed deer

The overall observed prevalence of *T. cervi* in adult white-tailed deer including resampled individuals was 65.9% (62/94; see Table 1). The overall prevalence of *T. cervi* in adult wild deer was 97.6% (41/42), which was significantly higher (*P* < 0.0001) compared to the prevalence (40.4%; 21/52 without resampling) in farmed deer *via* Fisher’s exact test (OR = 0.02; CI: 0.0004–0.117).

**Table 1:** *Theileria cervi* prevalence in adult white-tailed deer in Gadsden County, Florida, USA, spring and autumn 2016

	Wild		Farmed	
	No. positive/total no.	% positive (95% CI)	No. positive/total no.	% positive (95% CI)
Spring 2016	12/12	100 (75.8–100)	17/51	33.3 (22.0–47.0)
Autumn 2016	29/30	96.7 (83.3–99.4)	17/44	38.6 (25.7–53.3)
Total	41/42	97.6 (87.7–99.6)	21/52^a^	40.4 (28.16–53.9)

^a^This total value comes from 52 unique individuals that were tested overall in the two time periods, 51 of which were tested in spring 2016. 43 of those individuals were retested in autumn 2016 as well as an additional unique individual, which tested positive. The total number positive comes from 17 individuals that tested positive in spring and autumn, the 1 unique individual from autumn, and 3 female white-tailed deer that converted from negative to positive for *Theileria* DNA between spring and autumn

*Theileria cervi* infection prevalence in farmed deer sampled in spring 2016 was 33.3% (17/51). In autumn 2016, 43 of these farmed deer were re-sampled as well as an additional unique individual. The autumn prevalence for farmed deer was 38.6% (17/44). Three farmed females that tested negative in spring tested positive in the autumn, resulting in a final prevalence of 40.4% (21/52) for farmed, adult deer. Focusing only on farmed deer that we were able to resample, we calculated an incidence of infection rate of 3 incident cases/43 individuals/6 months. We found no significant difference in *T. cervi* prevalence between the farmed deer re-sampled in spring *vs* autumn (Fisher’s exact test, *P *= 0.65). Wild deer sampled in spring 2016 had a 100% (12/12) prevalence for *T. cervi* infection, and wild deer sampled in autumn 2016 had a 96.7% (29/30) prevalence. No wild deer were re-sampled in the autumn sampling period.

### Age as a predictor of infection

**Fig. 1:**
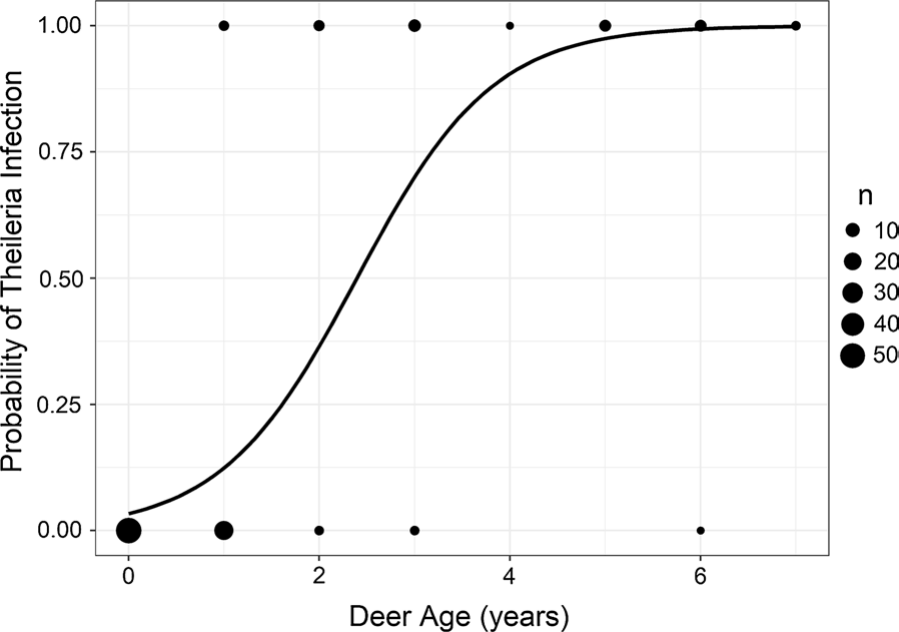
Probability of *Theileria cervi* infection with age as a predictive variable. Age was a positive, significant predictor variable of *T. cervi* infection in farmed white-tailed deer (*n* = 106)

None of the 53 fawns that were sampled at birth tested positive for *T. cervi*. Age was a significant predictor (OR = 4.06; CI: 2.37–6.98; *P *< 0.0001) of detection of *T. cervi* infection in farmed deer using logistic regression, with a logit equation of (y) = 1.402x − 3.359 and a McFadden *R*^2^ = 0.543 (Fig. 1). We found no significant difference in age between farmed (mean of 2.6 years) and wild (mean of 2.9 years) deer populations in our study (*t*_(74)_ = 0.34, *P* = 0.34). However, there were significant differences (*t*_(38)_ = -6.01, *P* < 0.0001) in the average age between farmed males (*n = *20) and females (*n* = 32) sampled (males, mean of 1.15 years; females, mean of 3.56 years). We were therefore unable to determine if there were differences in prevalence between the sexes of farmed deer due to the confounding effects of age. Given that all wild deer except one tested positive for *T. cervi*, there were no observable differences between the sexes for prevalence of *T. cervi* infection in wild deer (OR = 0; CI: 0–184.7; *P = *1.0).

### Genotypic variation

**Fig. 2:**
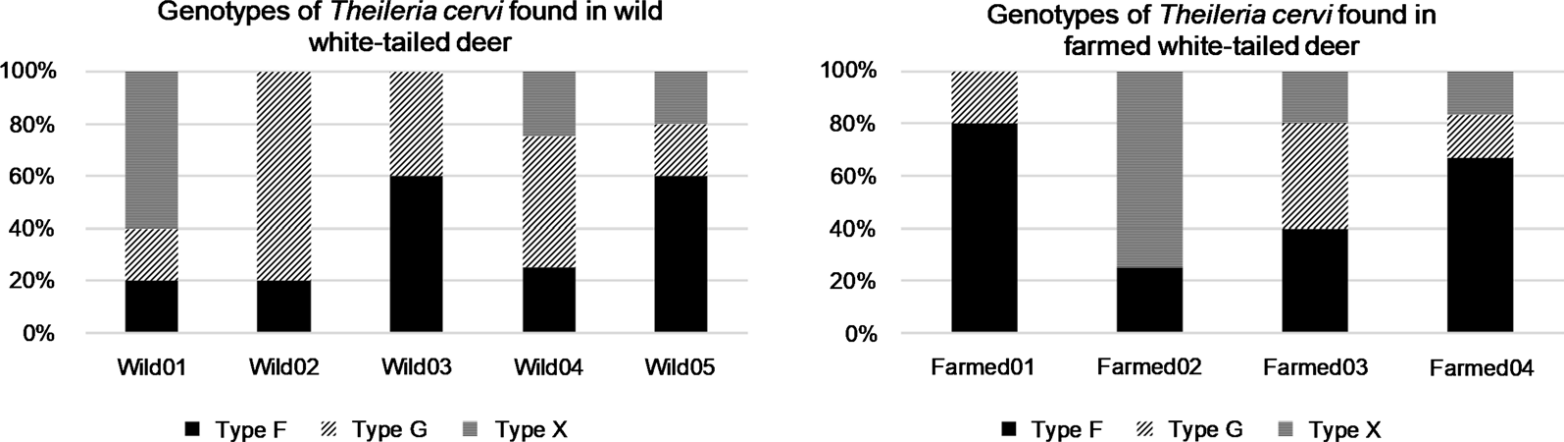
Genotypic variation of *Theileria cervi* between wild and farmed cervids. *Theileria cervi* clones found in wild (left) and farmed (right) white-tailed deer in Gadsden County, Florida, 2016. This figure illustrates the proportion at which each genotype was found infecting individual deer. Genotypes F, G, and X were present in both populations, and there were no significant differences in proportion of genotypes among treatments

**Fig. 3:**
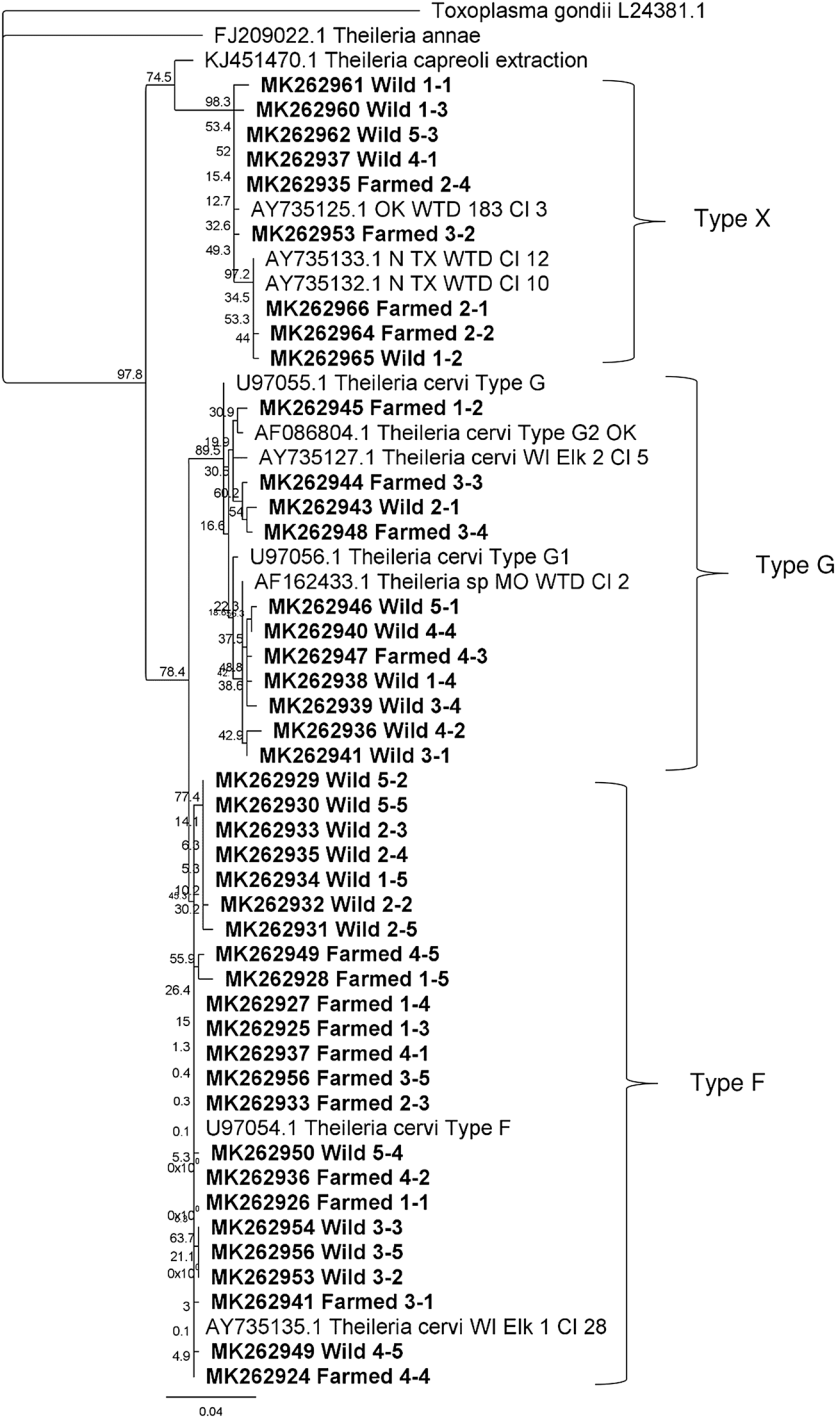
Phylogenetic analysis of *Theileria cervi* isolates in farmed and wild cervids. Maximum Likelihood phylogenetic tree generated from the partial DNA sequences of the V4 variable region of the small ribosomal subunits of the *Theileria* found in Florida farmed and wild white-tailed deer in this study (shown in bold) and closely related *Theileria* sequences isolated from cervine species in northern Texas, Wisconsin and Oklahoma, obtained from the NCBI database (GenBank accession numbers are shown). Previously established *Theileria cervi* Type F and Type G sequences are indicated, as well as the divergent genotype, referred to as Type X. Molecular distances were estimated using the Tamura-Nei model, and bootstrap support values are shown as percentages at the major nodes based on 1000 replicates. Phylogenetic distances are depicted by branch lengths that correlate with the number of substitutions inferred according to the scale shown

*Theileria cervi* genotyping was possible for 43 total clones representing sequences generated from five wild white-tailed deer and four farmed deer. All deer tested (*n* = 9), whether wild or farmed, were co-infected with multiple genotypes. The most prevalent *T. cervi* genotype was Type F, comprising 53.5% (23/43) of the clones sequenced. Genotype G represented 25.6% (11/43) of the genotypes sequenced (Table 2). Genotype X was closely related to an isolate previously identified as *T. cervi* (GenBank: AY735125.1), and represented 20.9% (9/43) of the clones sequenced (Table 2). All sequences were deposited into the GenBank database under the accession numbers MK262924-MK262966. The three genotypes were found in similar proportions within individuals among treatment herds (Fig. 2). There were no statistical differences (Fisher’s exact test, *P *> 0.05; Table 2) in the proportions of genotypes of *T. cervi* present in farmed *vs* wild white-tailed deer. A phylogenetic tree of the V4 hypervariable region of the *18S* rRNA gene revealed genotype clades and subclades of *T. cervi* from both farmed and wild populations (Fig. 3). One clade within the genotype X contained *T. cervi* from only wild deer; no unique subclades were found to have *T. cervi* from only farmed deer.

**Table 2:** Prevalence of *Theileria* genotypes in adult white-tailed deer in Gadsden County, Florida, USA, in spring and autumn 2016

Genotype	Farmed (*n* = 19)	Wild (*n* = 24)	Total (*n* = 43)	*P*-value^a^
	% Positive (*n*)	% Positive (*n*)	% Positive (*n*)	
Genotype F	57.9 (11)	50.0 (12)	53.5 (23)	0.364
Genotype G	21.1 (4)	29.2 (7)	53.5 (23)	0.200
Genotype X	21.1 (4)	20.8 (5)	20.9 (9)	1.000

*Notes*: Sample size represents the number of clones sequenced per treatment (Farmed *vs* Wild). A total of 4 farmed individuals and 5 wild individuals were sampled. The percentage represents the prevalence of each genotype within the farmed or wild cohort. Genotype G was the most prevalent genotype in white-tailed deer. Genotype F was found at higher proportion than genotype X in wild deer, but not in farmed deer. These differences were not significant

^a^Fisher’s exact test

### Mortality

We examined if infection with *T. cervi* was associated with increased mortality in farmed deer. We found that all-cause mortality was marginally lower in *T. cervi* positive farmed deer, but the test was not significant. There was a 37% (11/30) mortality rate among *T. cervi* negative animals, while animals that tested positive experienced 14% (3/21) mortality (OR = 3.5; CI: 0.83–14.5; *P* = 0.09). Theileriosis was not implicated as a cause of death in any of the animals. Necropsy revealed many causes of death, including mostly mixed bacterial infections and hemorrhagic disease; however, there were no observable patterns attributed to *T. cervi* infection or co-infections.

## Discussion

Results of our study suggested that the regular use of acaricides significantly suppressed the prevalence of *T. cervi* infections in adult farmed deer compared to an adjacent herd of wild deer. We found that wild deer had a nearly a 100% infection rate of *T. cervi*, which was significantly higher than in farmed deer, even though farmed deer lived at high densities conducive to a high infection rate. This difference in prevalence was likely due to the combined use of oral and injectable ivermectin to reduce the number of tick bites an individual received, and the use of permethrin to reduce tick abundance in the environment, although we did not directly test for this. This difference in prevalence was not confounded by the age of the animals sampled. Despite the reduced prevalence in farmed *vs* wild deer, we found evidence of ongoing transmission in farmed animals, as indicated by three farmed does that tested positive for infection in the autumn when they were initially negative the previous spring.

Although our study was not designed to determine whether *T. cervi* infection was chronic or if animals cleared the infection, our serial observations support the hypothesis that infected animals remain infected for at least six months. In addition, we found that older animals were more likely to be infected than younger animals, which would be expected for a vector-borne pathogen that establishes chronic infections in vertebrate hosts. These observations are thus in line with the general consensus that animals parasitized with *Theileria* usually do not clear their infections [8]. Furthermore, we found no evidence for vertical transmission of *T. cervi*, despite evidence for this mode of transmission in other *Theileria* spp. [22, 32]. No fawns that were sampled shortly after birth tested positive for *T. cervi*, suggesting that no vertical transmission occurred between dam and fawn. Additionally, we found no evidence of increased mortality for *T. cervi*-infected deer. This is most likely due to a long history of co-evolution between the hemoparasite and the vertebrate host [26], or an artifact of the short duration of the study.

We found multiple *Theileria* genotypes circulating in farmed and wild deer (Fig. 2). All *T. cervi* genotypes present in the farmed deer were found in the wild deer at roughly similar proportions. We did find evidence of one subclade of *Theileria*, genotype X, that was found only in wild deer. Because the sample sizes were small, it is difficult to determine if this is due to transmission barriers between the adjacent populations, the protective effects of acaricides, or a sampling anomaly. We cannot rule out that tick assemblages or tick habitat use differed. Currently, little is known about the significance of co-infection with different *T. cervi* genotypes, but co-infection has been identified as an important field of exploration, as many *Theileria* infections involve multiple genotypes [33]. All animals assessed in our study for genotypic variation in *T. cervi* were found to have mixed infections. Future experimental studies should explore whether different mixtures of genotypes of *Theileria* result in different or more severe illness.

While *T. cervi* infection is usually considered benign, it has been linked to illness in naïve animals or in animals subjected to different stressors, such as relocation or infection with other pathogens [12, 14, 34]. Fawns in particular are susceptible to the development of disease caused by *T. cervi* infection. The development of improved technologies such as vaginal implant transmitters has allowed researchers to gain a better idea of the causes of wild fawn mortality, and *T. cervi* infection might play a larger role in fawn mortality than previously thought [17]. Additionally, the relocation of species for farming or for other management scenarios can facilitate transmission of vector-borne diseases to new geographical areas, particularly when the receiving population is naïve to the pathogen [2]. More work is needed to determine which regions are endemic for *T. cervi* so informed decisions can be made regarding movement of animals. Additionally, the stress of relocation may result in immunosuppression and increase an animal’s susceptibility to infection. In order to avoid cases of theileriosis, we recommend that farmers reduce additional stressors such as overstocking pens.

*Theileria cervi* has been demonstrated to infect additional cervid species, including elk and mule deer, although the disease impact is not well studied. Game farms often keep multiple deer species on site, and the potential for transmission between species cannot be ruled out. Documented instances of transmission of *T. cervi* infection between species is limited; however, when the receiving deer is naïve to the pathogen, it can result in rapid morbidity and mortality. Farmers should consider pest management strategies that reduce tick abundance in the environment as well as treatment strategies that reduce overall parasitemia of infected deer (such as administration of buparvaquone) in order to reduce the risk of parasite transmission between species, particularly when animals are being transported to new regions.

This study demonstrates that acaricides do have measurable success at reducing the burden of tick-borne disease on farmed white-tailed deer during summer months. The farm on which this study was conducted treated animals and the environment for arthropod vectors as well as for endo- and ectoparasites, and represents a management regime that aggressively uses chemical control. Nonetheless, infection with *T. cervi* on the farm remained relatively high (~40%) and the nearly 100% infection rate of nearby wild deer suggests that the risk of infection is high in this geographical region. While exposure to *T. cervi* did not appear to have negative health effects on this cervid livestock species, neonates may be particularly at risk due to their susceptibility to disease [17]. If further tick control is needed to reduce tick populations and resultant tick-borne disease on these farms, acaracide application during spring or autumn may decrease the risk infection, but the increased exposure of deer to chemical vector control agents should be taken into consideration. In addition to acaracide use, integrated pest management strategies (e.g. prescribed burning, biological control, antiprotozoal medication) should be implemented.

## Conclusions

Overall, there is a greater need to understand how the use of acaricides on deer farms is impacting *T. cervi*, other vector-transmitted agents, and white-tailed deer health. In Florida and elsewhere, deer are infected with epizootic hemorrhagic disease virus that is transmitted by *Culicoides* midges [35], and deer can also be infected with *Plasmodium odocoilei*, or deer malaria, believed to be transmitted by *Anopheles* mosquitoes [36]. Both diseases affect morbidity and mortality of deer [37] and may impact their susceptibility to other vector-borne diseases. Little is known of the ecology of other vector-borne pathogens infecting white-tailed deer in Florida or how alternative management regimes may affect their epidemiology. Our study has shown that the prevalence of a tick-borne pathogen, *T. cervi*, is reduced using acaricides and is more prevalent in wild populations of white-tailed deer. Additionally, *T. cervi* is diverse within populations and individuals.

## Abbreviations

EDTA: Ethylene diamine triacetic acid
FWC: Florida Fish and Wildlife Commission
IACUC: Institutional Animal Care and Use Committee
